# Development and Preliminary Validation of Refugee Trauma History Checklist (RTHC)—A Brief Checklist for Survey Studies

**DOI:** 10.3390/ijerph14101175

**Published:** 2017-10-04

**Authors:** Erika Sigvardsdotter, Henrik Nilsson, Andreas Malm, Petter Tinghög, Maria Gottvall, Marjan Vaez, Fredrik Saboonchi

**Affiliations:** 1 Department of Health Sciences, The Swedish Red Cross University College, 14121 Huddinge, Sweden; henrik.nilsson@rkh.se (H.N.); andreas.malm@rkh.se (A.M.); petter.tinghog@rkh.se (P.T.); maria.gottvall@rkh.se (M.G.); Fredrik.saboonchi@rkh.se (F.S.); 2 Division of Insurance Medicine, Department of Clinical Neuroscience, Karolinska Institutet, 17177 Stockholm, Sweden; marjan.vaez@ki.se; 3 Swedish Red Cross Center for Persons Affected by War and Torture, 20121 Malmö, Sweden; 4 Department of Public Health and Caring Sciences, Uppsala University, 75236 Uppsala, Sweden; 5 Department of Women’s and Children’s Health, Uppsala University, 75236 Uppsala, Sweden

**Keywords:** refugees, trauma, population study, trauma checklist, self-report instrument

## Abstract

A high proportion of refugees have been subjected to potentially traumatic experiences (PTEs), including torture. PTEs, and torture in particular, are powerful predictors of mental ill health. This paper reports the development and preliminary validation of a brief refugee trauma checklist applicable for survey studies. *Methods*: A pool of 232 items was generated based on pre-existing instruments. Conceptualization, item selection and item refinement was conducted based on existing literature and in collaboration with experts. Ten cognitive interviews using a Think Aloud Protocol (TAP) were performed in a clinical setting, and field testing of the proposed checklist was performed in a total sample of *n* = 137 asylum seekers from Syria. *Results:* The proposed refugee trauma history checklist (RTHC) consists of 2 × 8 items, concerning PTEs that occurred before and during the respondents’ flight, respectively. Results show low item non-response and adequate psychometric properties *Conclusions:* RTHC is a usable tool for providing self-report data on refugee trauma history surveys of community samples. The core set of included events can be augmented and slight modifications can be applied to RTHC for use also in other refugee populations and settings.

## 1. Introduction

A large share of refugees has been subjected to potentially traumatic experiences (PTEs), including torture; approximately 20–40 percent of non-clinical samples of refugee groups report having experienced torture [1]. PTEs, and especially torture, are powerful predictors of mental ill health, particularly post-traumatic stress disorder (PTSD) symptoms, depression and anxiety [2,3] and somatization [4]. Given the significance of mental ill health, assessment of refugees’ trauma history emerges as utterly important, not merely as pertaining to individual subjective suffering but also as relating to a range of social issues and agendas [5].

Reports of the prevalence of refugee trauma are generally found as part of a broader analysis of refugee mental health, where trauma is measured and used as a background variable [1]. Furthermore, the majority of studies in this field are based on small convenience or consecutive samples, recruited in various community or clinical contexts [6]. The Harvard Trauma Questionnaire (HTQ) part 1 [7] is the most commonly used instrument to measure pre-migration PTEs in non-clinical samples of refugees [6]. Despite being developed in a clinical context, it has been described as a research standard for this group [8]. Among the trauma checklists used with refugees in non-clinical samples, only three—the Comprehensive Trauma Inventory (CTI) [9], the Communal Traumatic Events Inventory (CTEI) [10] and the HTQ part 1 [7]—are developed specifically for adult refugee groups. Two of these were developed in a clinical context. Other measures used are either developed in relation to general populations in Europe and North America or in relation to childhood refugee trauma [6].

Measuring refugee trauma history in large-scale population studies requires consideration of typical trauma backgrounds, language and culture specific adaptation of items and instruments [11,12], as well as assessment tools adapted to the methodological conditions of population studies and survey data collection methods. Structured interviews in a clinical context give other options for support and building of trust than does population-based mail or internet-based surveys. Questions concerning traumatic events can be perceived as sensitive and intrusive. Minimizing intrusiveness in the wording of such questions in surveys is of importance for both ethical reasons and for reducing low response rates or non-response [13].

The purpose of this study is to develop a refugee trauma-checklist suited for administration as a self-report measure in survey studies. This article reports the development and preliminary validation of the Refugee Trauma History Checklist (RTHC), fitted for questionnaires and surveys of adult refugees and asylum seekers.

## 2. Materials and Methods

Conceptualization of war and refuge related PTEs was based on existing research literature on refugee trauma as PTEs related specifically to the refugee experience, including war, conflict, persecution and flight/migration. The initial item pool of 232 trauma items was generated from existing trauma checklists used with adult refugees. These checklists are: the CTEI [10], the CTI [9], the HTQ part 1 [7], the Posttraumatic Stress Diagnostic Scale (PDS) part 1, [14], the Stressful Life Events Screening Questionnaire (SLESQ) [15], Traumatic Life Events Questionnaire (TLEQ) [16], War Trauma Questionnaire (WTQ) [17] and the War Trauma Scale (WTS) [18]. For an overview, see Sigvardsdotter et al. [6].

Item selection, as well as item refinement and wording, were performed by the research group in collaboration with experienced clinical psychologists with expertise in refugee war and torture trauma. During the review of the item pool items that were peripheral to the refugee experience (e.g., sexual contact as a child, partner abuse), were removed, as were items deemed too sensitive given the context of a mail survey, or containing possibly sensitive information (e.g., forced to injure or kill someone) or too specific (e.g., saw and touched dead bodies apart from funerals) or using culture specific metaphors (e.g., brainwashing).

Where conceptually possible, broader categories of traumatic events or situations were constructed. All kinds of physical, non-sexual assault were merged into one item “physical violence”. Rape and any other sexual assault were merged into “sexual violence”, thus avoiding using the highly charged word “rape”. Finally, a list of eight trauma items was arrived at. The items were posed twice; once referring to the period before leaving home, and once referring to the period after leaving home, with separate instructions regarding these differing contexts (see Appendix A).

In order to lessen the intrusiveness of the questions, rather than posing each question as a personal experience, an overall initial question “Have you experienced any of the following events or situations” was formulated, followed by a list of items. The trauma items were listed with a check box in front of each item.

The RTHC was translated into Arabic. Standard double-blind translation and back-translation procedures were used.

The RTHC items and the introductory question (see Appendix A), as well as a Single General Trauma Item (SGTI) also included in the questionnaire (see Appendix B), were discussed in a reference group of Syrian refugees with expertise in mental health research, psycho-social support and/or Arabic language. The reference group were met with twice. First, cultural aspects of what may be too sensitive to ask, as well as issues relating to appropriate data collection methods were discussed. The second focus group discussion was focused on language, making sure that translations were both linguistically and culturally appropriate.

To measure content validity, the clinical health professionals active at Swedish Red Cross Centers for Persons Affected by War and Torture (who had not previously been involved in the development of the checklist) were contacted via email and asked to rate the relevance of the 16 (8 × 2) items included in relation to the construct definition (see above). They rated each item on a four-point scale: “not relevant” (0), “somewhat relevant” (0), “quite relevant” (1), and “highly relevant” (1). A Content Validity Index for each item (I-CVI)—the proportion of the experts rating an item quite or highly relevant—was calculated, as well as the average I-CVI for the entire scale (S-CVI). While recommendations vary, an S-CVI of 0.9 or more should be strived for [19].

Usability of the RTHC was tested in a cognitive interview (CI) procedure [20] at a rehabilitation center for war and torture trauma patients. Consecutive patients (when the researchers were on duty) indicating Arabic as mother tongue were asked to participate. A convenience sample of *n* = 10 patients was included. CIs are valuable in pre-testing questions that are complex, sensitive or intrusive as well as questions that are planned to be used for specific groups that are expected to experience difficulties with completing the questionnaire [20]. The CIs were conducted by an experienced clinician and an experienced interpreter. The interviewer instructed the interview persons to read the questions out loud and to follow a Think-Aloud Protocol (TAP) [21] which means that they tell the interviewer everything they are thinking while filling in the RTHC. TAP is used to provide information about potential difficulties that may arise due to problems with comprehension, memory retrieval, judgment and response formatting on each item [20]. Upon any indication of such difficulties, the target item was further scrutinized by the research group, language experts, and by examining the patterns of data from field testing.

Initial field testing was carried out in a total sample frame of 373 persons from Syria registered as living in an accommodation for asylum seekers in Sweden. The data collection was done within the realm of a larger research project approved by the Regional Ethics Board, Stockholm (ref no.: 2015/1463-31). The data collection included a larger set of self-report instruments (see below). The questionnaires were distributed by “questionnaire guides”, i.e., volunteers among the asylum seekers who had received training in survey methods and psycho-social support. They were also available to assist the study participants with the questionnaire if needed. Of the total sample frame, 56 persons could not be located despite repeated visits, and were assumed to have left the center. Thus, the questionnaire was distributed to *n* = 317, together with envelopes with pre-paid postage, by which they could be anonymously returned.

In addition to RTHC, the first 16 trauma symptom items of the HTQ part IV, was included in the questionnaire. The instrument measures symptoms of PTSD during the last week, using four response alternatives ranging from “not at all” (1) to “very much” (4). Mean item scores were calculated.

Due to lack of a gold standard for PTEs, a single general trauma item (SGTI) was formulated broadly in accordance with International Classification of Diseases, ICD-10 diagnostic criterion A for PTSD (F 43.1) [22] and included in the questionnaire. Using the SGTI as key variable, convergent validity of each item of RTHC was assessed by estimating the binominal phi correlation coefficient (Φ). Convergent validity is the extent of association between two measures of constructs that are assumed to be related [23]. To acquire further measures of associations between each item of RTHC and mental ill health outcomes, the PTSD scores of HTQ part IV were compared between those endorsing and those not endorsing each RTHC item by independent *t*-tests. Due to multiple significant testing within each section of RTHC, the *p*-value for all analyses was adjusted by the Bonferroni method [24] and set at α = 0.006. All analyses were performed using IBM SPSS 24 (IBM, Armonk, NY, USA).

## 3. Results

### 3.1. Content Validity

Seven clinicians rated the relevance of the items on the RTHC checklist. The I-CVI for all items was 1.00, as was consequently the S-CVI. Only “witnessing physical violence” and “other frightening situation where you felt your life was in danger” were rated “quite relevant” by one expert, otherwise all were considered “highly relevant”.

### 3.2. Cognitive Interviews

Of the 10 interview persons, seven were male and three female of varying ages (23–59, average 45 years old). Their educational backgrounds varied, from 9 years of schooling to university degrees. They originated from different countries: Iraq (*n* = 7), Palestine (*n* = 1) and Syria (*n* = 2), and had resided in Sweden between 3 and 36 years (average 10 years).

The results of the TAP indicated some potential difficulties regarding comprehension of the Arabic translation. The word “home” raised questions whether it meant home or home country. Further, the word “forced” (in forced separation) rendered questions as potentially implicit in the concept of displacement, since fleeing home involves separation from relatives and friends. All interview persons were, however, able to provide a response to the items containing these words. 

Some minor difficulties were indicated regarding judgment of the time-period implied by the wording “during the flight”, as it could mean the flight within their home country as well as time spent in refugee camps or other places they stayed for longer periods of time. Similar minor difficulties were indicated for item “war at close quarters” which prompted questions concerning “how close” this meant. Despite these difficulties, all the interview persons arrived at an answer that they found appropriate and could respond to the items accordingly.

The questions raised in the CIs were discussed with a bilingual expert, and the overall assessment was that no modification was necessary.

The wording “sexual violence” prompted questions concerning its definition, as well as comments on its sensitive nature. After discussions with bilingual community experts it was decided that further specification of the item would result in rendering the wording too intrusive.

Although the CIs did not result in modification of any items, the pattern of dispersion, as well as item non-response rates in the field testing were closely examined in relation to the items indicated by CIs.

### 3.3. Field Testing 

In total, *n* = 137 returned the questionnaire, representing a 43.0% response rate. Of the respondents, 107 (78.7%) were asylum seekers, while the rest (21.3%) had recently received residence permit in Sweden but had not yet left the asylum residential center, 106 (77.9%) were men, and a majority (93.4%) had Syrian citizenship, the rest were stateless Palestinians born in Syria.

The frequencies of the various types of traumatic events can be found in Table 1. Before the flight, “War at close quarters” was the most common type of specific event/situation experienced (98.5%), followed by “Forced separation from family or close friends” (77.6%) and “Loss or disappearance of family member(s) or loved one(s)” (70.9%). During the flight, “Forced separation from family or close friends” (61.5%) and “Loss or disappearance of family member(s) or loved one(s)” (51.2%) were the most common. Of the respondents, 25.0% indicated that they had been tortured before leaving home, and 17.1% during their flight. Figures for torture were higher among men than women. Further, the “Other…” category, both before (92.4%) and during the flight (64.1%), was endorsed by a large proportion of respondents. Mean number of types of trauma before and during the flight was 4.92 (SD = 1.68). Item non-response varied between 0.7% and 6.6% (Table 1). The SGTI was endorsed by 62.5% (*n* = 85) and had a non-response rate of 8.0% (*n* = 11).

Binominal correlation coefficients Φ for the items are displayed in Table 2. The single trauma items displayed a pattern of overall weak and non-significant correlations after Bonferroni adjustment of *p-*value*s*, with the exception of “Witnessing physical violence or assault” in the *Before the flight* section, and “Other frightening situations…” in both sections. 

There was an overall pattern of significant differences in regard to PTSD symptom scores between endorsers and non-endorsers on RTHC items (see Table 3).

## 4. Discussion

The purpose of this study was to develop the RTHC, a refugee trauma-checklist adapted to data collection formats that do not offer the supportive and facilitating conditions of face-to-face clinical settings. Further, we gathered preliminary evidence of validity and usability of the Arabic version of the RTHC. The choice of Arabic for the linguistic adaption was due to the fact that Arabic speaking refugees currently comprise the largest refugee group in Sweden and the EU. Our preliminary results suggest that RTHC is a usable and valid tool with overall adequate properties, which with slight modifications can be linguistically and contextually adapted for use also in other refugee populations.

The sensitivity inherent in self-reporting of traumatic events poses several important methodological and ethical challenges. Sensitive contents in survey methodology are usually addressed in terms of intrusiveness to the respondents’ privacy, such as questions eliciting perceived threats of undesirable consequences if answered truthfully, or requiring answers that are socially unacceptable [13]. Beyond the risk of non-response, other ethical concerns such as the risk for raising the respondents’ expectation of receiving more information and support in potentially anxiety evoking questions [25] should also be considered. More specific to RTHC, recalling memories of exposure to traumatic events may be accompanied with negative emotional arousal [26].

In the present study, these issues were addressed by attempting to establish appropriate wording of the items [27], as well as by constructing broader categories [13] of trauma as opposed to detailed descriptions of traumatic events that may be too distressing. Further, we provided a support-line for respondents who wished to contact the research team during the survey [25]. This support-line was, however, very little used by the respondents. The overall low rates of non-response, high dispersion of responses, and low usage of the support-line, as well as the results of the cognitive interviews, indicate that the sensitivity of the included items do not pose a substantive threat to the overall checklist’s validity. An exception to this general pattern was, however, the item “sexual violence”. The very low endorsement of this item may either be ascribed to an actual low prevalence, or to the item dealing with a strong taboo. In either case, this is in accordance with earlier research [1]. Despite the inevitable sensitivity pertaining to the item “sexual violence”, it was decided that the item should remain on the checklist. Not including this category would exacerbate the invisibility of this type of violence and further reproduce the taboo, both with respondents and in research.

Regarding convergent validity of the included items in RTHC, an overall pattern of weak correlations with the SGTI was observed. These results can be seen as indications that the RTHC items, which target exposure to specific events, and a general trauma item based on the expected subjective emotional reaction to potentially traumatizing event, assess phenomena that vary independently. As such, these weak correlations may suggest discriminant validity [23] of these two constructs and modes of assessment. The pattern may be further explained by the fact that endorsing an included item in RTHC is predictive of having been exposed to a potentially traumatizing event; non-endorsement of a single RTHC item does not necessarily indicate that the respondent has not been exposed to a potentially traumatizing event, as exposure to one trauma does not exclude the possibility of exposure to another. Thus, the information provided by the RTHC items appears to be related but distinct from the information provided by a broad general question regarding emotional reactions to exposure to potentially traumatizing events.

The relevancy of the included items for mental health outcomes is, however, indicated by the observed general pattern of higher levels of PTSD symptoms among the endorsers of most of the items in the checklist, especially those related to violence. The lack of statistically significant differences in regard to some of the items, i.e., sexual violence, can be attributed to the small number of endorsers of this item. The opposite, i.e., small number of non-endorsers, applies to the non-significant result for “War at close quarters” in the *Before the flight* section.

Given the intended use of RTHC in surveys, the primary considerations in the development process were the range, content, as well as the wordings of the included traumatic events. As in similar assessments, the included items represent only a sample from a larger population of possible traumatic events [28]. As RTHC is constructed specifically for refugee populations, the included items in the list should be viewed as a core set of events that are relevant for and currently prevalent among refugees. In our field test, a substantive proportion of the participants (92.4%) endorse the item “Other frightening situation where you felt your life was in danger”, in the *Before the flight* section, while this proportion in the *During the flight* section was markedly lower (64%). This indicates that several other types of events occurring at the site of the conflict and persecution are not addressed in the list, while the included core set of items covers the possible events during the flight to a somewhat larger extent.

The included items, however, received overall high CVIs and showed varying patterns of endorsement, which points to relevancy of, and an adequate degree of differentiation by, this core set of items. Researchers intending to use RTHC are, however, encouraged to consider addition of population and context relevant categories of traumatic events to the list. 

Limitations: A main limitation of the present study is the unavailability of a gold standard for exposure to traumatic events in the targeted population. This lack of gold standard, which also constitutes the main motive for development of the present checklist, inevitability affects the overall process of development and validation. Although we used a very broad and general item to accommodate for the individual and subjective variations of reaction to exposure to traumatic events, this important limitation needs to be addressed in future studies in which a more precise and reliable gold standard for exposure to trauma, such as clinical interviews, may be available. Another limitation in the study was that measures of test-retest could not be provided because of the settings of the data collection, which only enabled the research group to make a single assessment. Further, and given the specific uncertainty surrounding asylum seekers’ conditions, asking the respondents these questions twice may have potentially conveyed that their responses could not be trusted, which would have been ethically undesirable. Consequently, we decided to not include measures of stability. The latter can, however, be provided in future studies conducted in more appropriate settings.

## 5. Conclusions

The reported research concludes that RTHC is a usable tool for providing self-report data on refugee trauma history surveys of various types of community samples. Previous checklists have been developed and validated in clinical samples, and thus may not be specifically suited for self-report surveys. The core set of included events can be augmented and slight modifications can be applied to RTHC for use also in other refugee populations and settings.

## Figures and Tables

**Table 1:** Prevalence of PTEs and non-response rates for each item among the respondents.

Various Types of Traumatic Events	Women *n* (%)	Men *n* (%)	Men and Women *n* (%)	Item Non-Response *n* (%)
**Before the Flight**				
War at close quarters	28 (93.3)	105 (100.0)	133 (98.5)	1 (0.7)
Forced separation from family or close friends	21 (70.0)	83 (79.8)	104 (77.6)	2 (1.5)
Loss or disappearance of family member(s) or loved one(s)	19 (63.3)	76 (73.1)	95 (70.9)	2 (1.5)
Physical violence or assault	4 (13.8)	38 (37.6)	42 (32.3)	6 (4.4)
Witnessing physical violence or assault	15 (51.7)	76 (73.8)	91 (68.9)	4 (3.0)
Torture	4 (13.8)	29 (28.2)	33 (25.0)	4 (3.0)
Sexual violence	3 (10.3)	3 (2.9)	6 (4.6)	5 (3.7)
Other frightening situation where you felt your life was in danger	25 (86.2)	97 (94.2)	122 (92.4)	4 (3.0)
**During the Flight**				
War at close quarters	12 (40.0)	37 (36.6)	49 (37.4)	5 (3.7)
Forced separation from family or close friends	15 (53.6)	65 (63.7)	80 (61.5)	6 (4.4)
Loss or disappearance of family member(s) or loved one(s)	14 (50.0)	52 (51.5)	66 (51.2)	7 (5.1)
Physical violence or assault	3 (10.7)	21 (21.0)	24 (18.8)	8 (5.9)
Witnessing physical violence or assault	6 (21.4)	40 (40.4)	46 (36.2)	9 (6.6)
Torture	4 (13.8)	18 (18.0)	22 (17.1)	7 (5.1)
Sexual violence	3 (10.7)	4 (4.0)	7 (5.4)	7 (5.1)
Other frightening situation where you felt your life was in danger	16 (55.2)	68 (66.7)	84 (64.1)	5 (3.7)

**Table 2:** Binominal correlation coefficients Φ and corresponding *p*-values of the items of RTHC with the SGTI as key variable.

Trauma Items	Φ	*p*
**Before the Flight**		
War at close quarters	0.17	0.05
Forced separation from family or close friends	0.03	0.74
Loss or disappearance of family member(s) or loved one(s)	0.24	0.007
Physical violence or assault	0.24	0.007
Witnessing physical violence or assault	**0.51**	**0.001**
Torture	0.09	0.29
Sexual violence	0.08	0.36
Other frightening situation where you felt your life was in danger	**0.35**	**0.001**
**During the Flight**		
War at close quarters	−0.02	0.80
Forced separation from family or close friends	−0.09	0.32
Loss or disappearance of family member(s) or loved one(s)	−0.03	0.71
Physical violence or assault	0.17	0.054
Witnessing physical violence or assault	0.18	0.046
Torture	0.12	0.17
Sexual violence	0.11	0.23
Other frightening situation where you felt your life was in danger	**0.31**	**0.001**

Correlations in bold remained significant after Bonferroni correction of α (*p* < 0.006).

**Table 3:** Means, standard deviation, and independent *t*-test of the differences in regard to PTSD symptom scores for endorsers and non-endorses of RTHC items.

Trauma Items	Yes		No		*t*	*p*
	M (SD)	*n*	M (SD)	*n*		
**Before the Flight**						
War at close quarters	1.94 (0.6)	131	2.15 (0.31)	2	0.50	0.62
Forced separation from family or close friends	**2.02 (0.60)**	103	**1.68 (0.52)**	30	2.84	0.005
Loss or disappearance of family member(s) or loved one(s)	**2.10 (0.56)**	94	**1.58 (0.52)**	39	4.97	0.001
Physical violence or assault	**2.27 (0.62)**	42	**1.79 (0.53)**	88	4.57	0.001
Witnessing physical violence or assault	**2.12 (0.58)**	91	**1.58 (0.46)**	41	5.17	0.001
Torture	**2.20 (0.71)**	33	**1.86 (0.53)**	*99*	2.91	0.004
Sexual violence	2.29 (1.02)	6	1.93 (0.57)	125	1.45	0.15
Other frightening situation where you felt your life was in danger	**1.99 (0.59)**	122	**1.44 (0.54)**	10	2.84	0.005
**During the Flight**						
War at close quarters	**2.13 (0.64)**	48	**1.83 (0.55)**	82	2.86	0.005
Forced separation from family or close friends	**2.08 (0.63)**	79	**1.70 (0.45)**	50	3.69	0.001
Loss or disappearance of family member(s) or loved one(s)	2.07 (0.61)	64	1.79 (0.56)	63	2.73	0.007
Physical violence or assault	2.23 (0.59)	24	1.86 (0.58)	104	2.86	0.005
Witnessing physical violence or assault	2.13 (0.63)	46	1.81 (0.55)	81	2.97	0.004
Torture	2.27 (0.81)	21	1.86 (0.52)	107	2.99	0.003
Sexual violence	2.24 (0.94)	7	1.91 (0.57)	122	1.43	0.15
Other frightening situation where you felt your life was in danger	**2.05 (0.61)**	83	**1.71 (0.50)**	47	3.30	0.001

M = Mean, SD = Standard Deviation; Mean values in bold remained significantly different after Bonferroni correction of α (*p* < 0.006).

## Appendix A

**The Refugee Trauma History Checklist**

The questions in this section concerns difficult and frightening experiences, and can awaken distressing memories. It is important for us that many people answer these questions. However, if you find it is too distressing, please take a break or skip this section.

***Before you left your home,* have you experienced any of the following situations or events?**	**Y/N**
War at close quarters	Y/N
Forced separation from family or close friends	Y/N
Loss or disappearance of family member(s) or loved one(s)	Y/N
Physical violence or assault	Y/N
Witnessing physical violence or assault	Y/N
Torture	Y/N
Sexual violence	Y/N
Other frightening situation(s) where you felt your life was in danger.	Y/N

***After you left your home*, during your flight, have you experienced any of the following situations or events?**	**Y/N**
War at close quarters	Y/N
Forced separation from family or close friends	Y/N
Loss or disappearance of family member(s) or loved one(s)	Y/N
Physical violence or assault	Y/N
Witnessing physical violence or assault	Y/N
Torture	Y/N
Sexual violence	Y/N
Other frightening situation(s) where you felt your life was in danger.	Y/N

## Appendix B

**The Single General Trauma Item (SGTI)**

Sometimes things happen to people that would upset or frighten almost everyone. Examples of such difficult and frightening experiences are: being assaulted, or witnessing other people being hurt or killed.

	**Yes**	**No**
Have you experienced any of these or some other terrifying event(s)?		
